# Feasibility of using breath sampling of non-volatiles to estimate the prevalence of illicit drug use among nightlife attendees

**DOI:** 10.1038/s41598-022-24741-1

**Published:** 2022-11-24

**Authors:** Kristin Feltmann, Tobias H. Elgán, Michael Böttcher, Stefan Lierheimer, Sigurd Hermansson, Olof Beck, Johanna Gripenberg

**Affiliations:** 1 STAD, Stockholm Prevents Alcohol and Drug Problems, Stockholm, Sweden; 2 Centre for Psychiatry Research, Department of Clinical Neuroscience, Karolinska Institutet & Stockholm Health Care Services, Region Stockholm, Norra Stationsgatan 69, plan 7, 11364 Stockholm, Sweden; 3 MVZ Medizinische Labore Dessau Kassel GmbH, Dessau-Roßlau, Germany; 4 Waters Sverige AB, Solna, Sweden

**Keywords:** Health occupations, Biological techniques, Analytical biochemistry, Mass spectrometry

## Abstract

The prevalence of drug use among nightlife attendees needs to be accurately estimated to, for example, evaluate preventive interventions. This study tested the feasibility of using a breath-sampling device to estimate the prevalence of drug use among nightlife attendees. The study was conducted at five nightclubs and a large music festival in Stockholm, Sweden. Participants were invited to participate and microparticles in exhaled breath were sampled and analyzed for 47 compounds using a state-of-the-art analytic method that follows forensic standards. In addition, participants’ breath alcohol concentration was measured and they were interviewed about demographics, drinking habits, and drug use. Of the people invited, 73.7% (n = 1223) agreed to participate, and breath samples were collected from 1204 participants. Breath sampling was fast and well-accepted by participants. 13 percent of participants tested positive for an illicit drug, but only 4.3% self-reported drug use during the last 48 h. The most common substances detected were cocaine, amphetamine, and MDMA. There was no agreement between self-reported and measured use of any drug. Breath sampling is a convenient method to test illicit drug use among a large number of participants at events, and can be used as an estimate of drug use prevalence.

## Introduction

Young adults frequently visit and socialize in different nightlife venues such as bars, clubs, and festivals, and studies have shown that psychoactive substance use is especially high in these environments^1–11^. Research indicates that illicit drug use is associated with high levels of alcohol consumption^5,6,9,12^. The use of alcohol and illicit drugs can lead to serious acute consequences such as violence (including sexual assault), accidents, and even death^13–24^. Frequent use of these substances increases the risk of both physical and mental illnesses, including substance use disorders^13^. Several studies have shown that one in three visitors of music festivals is heavily alcohol intoxicated^10,25^. Furthermore, between 10 and 25% visitors of nightclubs and music festivals were tested positive for illicit drugs^4,5,26,27^. The fact that such a large number of visitors of nightlife events are alcohol- and drug-intoxicated involves not only risks for the individual but also safety risks with, for example, evacuations due to fires or terror threats^28–30^. Together, these substance-related consequences could potentially be prevented by implementing preventive measures. To assess the needs of such interventions as well as to evaluate their effects, appropriate methods to estimate substance use are required. By measuring substance use at several music events and clubs before and after the intervention has been implemented, both in the intervention and a control area, the effect of the intervention could be estimated.

To date, nightlife event attendees have mainly been interviewed about their drug use using face-to-face surveys or written questionnaires. However, a large proportion of drug users do not self-report drug use (i.e., underreporting), which has been revealed using biological testing^3,5,9,27^. Several biological specimens such as hair^31,32^, blood^8^, urine^8^, and, more commonly, oral fluid^3,5,8–10^ have been sampled among nightlife attendees to test drug use. Hair samples are not suitable for assessing recent drug use, given that the detection window is between several days to months or years after drug use and will vary across individuals due to different hair lengths. Moreover, not all participants were willing to provide a hair sample. Blood sampling requires certain equipment and training to be performed safely, and because of its invasiveness, it might be even less accepted by participants. Similarly, sampling urine requires toilet facilities and is unfeasible in nightlife settings. Several studies have shown that oral fluid sampling can be used to assess the prevalence of drug use among nightlife attendees and is superior to self-reporting^3,5,9^. Oral fluid samples in these studies were analyzed using mass spectrometry, a highly sensitive and specific method to analyze drug use off-site. For example, among Swedish attendees of a two-day electronic dance music (EDM) event on a cruise ship, 10% tested positive for illicit drugs, but only 4% had reported recent drug use^5^. Similarly, among Norwegian festival goers, 11% tested positive for illicit drugs, but only 6% had reported drug use^9^. Furthermore, matching self-report and drug testing results among club goers in the San Francisco Bay area revealed moderate agreement between the two measures^3^. In addition, an on-site immunological quick screening test of oral fluid samples has also been conducted among Australian club goers, demonstrating that 9% had reported drug use, and 20% tested positive^27^. Although oral fluid sampling is a non-invasive method, which seems to be in good agreement with the results obtained from blood sampling^8^, in some participants, sampling can be time-consuming, often due to a dry mouth, which can be induced by some drugs or medications^5^. More recently, studies have demonstrated that breath sampling can be used to detect a variety of illicit drugs and medications^33–35^; however, to our knowledge, this has not been tested in nightlife settings.

We aimed to investigate the feasibility of testing a large number of nightlife attendees for the recent use of illicit drugs by using a device that enables the quick and easy sampling of aerosol particles in exhaled breath^34^. More specifically, we tested whether breath sampling is well-accepted and if drug testing is superior to self-reporting for estimating the prevalence of recent drug use. Furthermore, we aimed to investigate the validity of self-reporting regarding the recent use of various substances by matching drug testing results at an individual level. We were also interested in comparing drug use prevalence, as well as the extent of underreporting between genders and the alcohol levels between people who tested positive and negative for drug use.

## Results

Of the 1659 persons who were invited to anonymously participate in the study, 436 refused, and 1223 agreed, leaving a response rate of 73.7%. Those who refused participation had been informed about the study's aim to investigate alcohol and drug use but had not yet been informed about drug testing. All participants agreed to alcohol breath testing, but breath samples for drug testing were not collected from 19 participants (1.6%). Of these, 12 participants dropped out when they were asked to complete a self-reported questionnaire on illicit drug use. Thus, these 12 participants dropped out before they were asked to conduct breath sampling. Only seven participants (0.6%) actively refused breath sampling. In general, the use of the device was well accepted, and most participants found the sampling procedure easy and fast (between 2 and 4 min, including instructions). Some participants started to exhale with much force or very slowly, in which case they were reminded to exhale normally. In particular, highly intoxicated participants sometimes had difficulties following the instructions.

### Demographics and past substance use

The majority of the participants were male (57.1%), the median age was 23 years (interquartile range: 20–27 years, missing n = 3), and the largest proportion was working full-time (Table 1). More than 80% reported that they visited clubs at least six times per year. Whereas more than half were non-smokers (tobacco), almost 11.6% were daily smokers. Furthermore, 78.2% of participants had a risky alcohol consumption, defined by an AUDIT-C score of at least five for men and four for women. Three out of ten (30.5%) of the participants reported having used illicit drugs during the last year and 46.4% had used cannabis at least once during their lifetime.

**Table 1:** Demographic data.

	% (95% confidence interval, CI), n
**Gender**	
Men	57.1 (54.3–59.9), 697
Women	42.5 (39.8–45.4), 519
Other	0.3 (0.1–0.8), 4
**Occupation**	
Full-time	65.1 (62.3–67.8), 796
Part-time	9.0 (7.5–10.7), 110
University student	18.5 (16.3–20.8), 226
High-school student	4.2 (3.1–5.5), 51
Unemployed	1.2 (0.7–2.0), 15
Other	2.0 (1.3–3.0), 25
**Number of club visits per year**	
< 6 times	17.0 (15.0–19.3), 204
≥ 6 times	83.0 (80.7–85.1), 993
**Recruited at nightlife setting**	
clubs (n = 5)	45.3 (42.5–48.1), 554
2-day festival	54.7 (51.9–57.5), 669
**Smoking**	
Non-smoker	55.9 (53.1–58.7), 681
Non-daily Smoker	32.5 (29.9–35.2), 396
Daily Smoker	11.6 (9.8–13.5), 141
**Alcohol habits during the last year**	
Abstainers	2.4 (29)
Non-hazardous drinkers	18.1 (220)
Hazardous drinkers	78.2 (956)
**Illicit drug use**	
Never	49.0 (593)
Several years ago	20.5 (248)
During the last year	30.5 (369)
**Ever use**	
Cannabis	46.4 (558)
Cocaine	21.7 (260)
Ecstasy/MDMA	19.7 (236)
Amphetamine	9.6 (115)

Participants, n = 1223. Hazardous drinkers are defined as having a score of at least 4 or 5 points in the Alcohol Use Disorders Identification Test-Concise (AUDIT-C) for women and men, respectively. Data is missing for n = 3 (gender), n = 26 (club visits), n = 5 (smoking), n = 8 (Alcohol habits), n = 13 (Illicit drug use), n = 20 (cannabis), n = 23 (cocaine), n = 22 (ecstasy), and n = 23 (amphetamine).

For people who refused participation, gender and age were estimated and recorded. The proportion of men was lower among participants than among non-participants (57.3% vs. 63.1% male, χ^2^(1) = 4.38, *P* = 0.036). Furthermore, the age of the participants was lower than that of non-participants (median age: 23 vs. 25 years, U = 225.4, n = 1,656, *P* < 0.001), although only the approximate age was estimated for the latter.

### Recent use of illicit drugs: self-reporting and biological testing

Analysis of the breath samples revealed that out of 47 substances analyzed, 22 were found in breath samples related to 19 different illicit drugs (Table 2). While 13.0% of the participants tested positive for at least one substance, only 4.3% had self-reported drug use during the past 48 h. The most common drugs found in the biological tests were cocaine, amphetamine, and 3,4-Methylenedioxymethamphetamine (MDMA). In the vast majority of the positive samples (88.5%, n = 138), one type of illicit drug was detected. 13 participants tested positive for two different drugs, and few tested positive for three (n = 3), four (n = 1), or five (n = 1) different drugs.

**Table 2:** Prevalence of the recent use of illicit drugs obtained by self-reporting and drug test results of exhaled breath samples (n = 1223).

	Self-reported use during the last 48 h % (95% confidence interval, CI), n	Positive test result of breath sample % (95 CI), n
Any illicit drug^1^	4.3 (3.2–5.6), 52	13.0 (11.1–15.0), 156
Polydrug use^2^	0.7 (0.3–1.3), 8	1.5 (0.9–2.4), 18
Cannabis/THC^3^	2.4 (1.6–3.5), 29	0.8 (0.4–1.5), 10
Cocaine	1.7 (1.0–2.6), 20	6.4 (5.1–7.9), 77
Ecstasy	0.6 (0.2–1.2), 7	1.8 (1.2–2.8), 22
Amphetamine	0.2 (0.1–0.7), 3	3.8 (2.8–5.1), 46
Mushrooms^4^	0.2 (0.0–0.6), 2	–
LSD^4^	0.2 (0.0–0.6), 2	–
NPS^5^	0.2 (0.0–0.6), 2	0.2 (0.0–0.6), 2
Ketamine	0.1 (0.0–0.5), 1	0.1 (0.0–0.5) 1
Heroin	0.1 (0.0–0.5), 1	0.2 (0.0–0.6), 2

^1^Self-reported drug use of at least one substance listed in the table. Positive test result of at least one of the 47 substances tested.

^2^Self-reported use of two or more drugs during the last 48 h by the same individual. Positive test results of two or more drugs within the sample.

^3^Analyses of breath samples can only detect cannabis used within approximately the last 6 h.

^4^Magic mushrooms and LSD were not tested for in the breath samples.

^5^Of the four compounds previously defined as new psychoactive substances (NPS), two were found in the breath samples: pentedrone (n = 2), alpha-PVP (n = 2), within the same two subjects. In addition, the following drugs were found in the samples: methylphenidate (n = 7), Methamphetamine (n = 1), MDA (n = 1), Oxazepam (n = 1), Temazepam (n = 2), Pregabalin (n = 2), Gabapentin (n = 1), Tramadol (n = 1), Oxycodone (n = 1), Zopiclone (n = 1), Methadone (n = 1).

Participants, n = 1,223. Regarding self-reported data, data was missing for n = 13 (any illicit drug, polydrug use), n = 34 (cannabis), n = 31 (cocaine), n = 29 (ecstasy), n = 21 (amphetamine), n = 16 (mushrooms, LDS, ketamine), and n = 18 (NPS, heroin). Breath samples were not obtained from 19 participants.

Significantly more men than women tested positive for illicit drugs (15.4% vs. 9.6%, χ^2^ (1) = 8.79, *P* = 0.003). Among men who tested positive for an illicit drug (n = 105), 21.0% had self-reported the use of illicit drugs during the last 48 h. Among women who tested positive (n = 49), 6.1% had self-reported the use of illicit drugs during the last 48 h.

There was no agreement between the results of breath sampling and self-reporting regarding drug use during the last 48 h (Table 3). For example, among participants who self-reported no recent drug use (n = 1149), 11.4% tested positive for at least one illicit drug. Among those who self-reported recent drug use, approximately half tested positive for illicit drugs. Concerning central stimulants, matching between self-reported drug use and biological testing was non-existent or minimal (Table 3), and several of the participants who had not self-reported recent use tested positive, indicating underreporting. Regarding cannabis, there was no match between self-reporting and biological testing. For instance, none of those who self-reported recent use tested positive, but this was attributed to the shorter detection time for tetrahydrocannabinol (THC) in exhaled breath. Moreover, none of the participants who tested positive for cannabis had reported recent use. The few subjects who tested positive for NPS, ketamine, or heroin (Table 2) had not self-reported use of the respective substances.

**Table 3:** Matching self-reported and measured drug use (n = 1201).

	Tested positive % (95% confidence interval, CI), n	Cohen's kappa
**Any illicit drug**		
Did not self-report (n = 1149)	11.4 (9.6–13.4), 131	0.188
Did self-report (n = 52)	48.1 (34.0–62.4), 25	
**Cannabis/THC**		
Did not self-report (n = 1151)	0.8 (0.4–1.5), 9	− 0.012
Did self-report (n = 29)	0	
**Cocaine**		
Did not self-report (n = 1163)	5.2 (4.0–6.7), 61	0.275
Did self-report (n = 20)	70.0 (45.7–88.1), 14	
**Ecstasy/MDMA**		
Did not self-report (n = 1178)	1.3 (0.7–2.1), 15	0.365
Did self-report (n = 7)	71.4 (29.0–96.3), 5	
**Amphetamine**		
Did not self-report (n = 1190)	3.7 (2.7–4.9), 44	0.077
Did self-report (n = 3)	66.7 (9.4–99.1), 2	

Data is missing for n = 22 (any illicit drug), n = 43 (cannabis), n = 40 (cocaine), n = 38 (ecstasy), and n = 30 (amphetamine).

Of all participants, 88.1% had a BAC level above 0%, with a median of 0.09% (interquartile range, 0.05–0.12%), and 35.5% of participants had a BAC level of 0.10% or above. Among participants who tested positive for illicit drugs, the BAC level was significantly higher than that among participants who tested negative for an illicit drug (median, interquartiale range): 0.094%, 0.52–0.13% vs. 0.075%, 0.35–0.12%, U = 92,562, *P* = 0.007).

## Discussion

Breath sampling was accepted well and could easily be collected from a large number of participants in different nightlife settings. The analysis of breath samples could detect the use of 19 different substances, most commonly cocaine, amphetamine, MDMA (ecstasy), and cannabis. Drug testing revealed that 13.0% of participants had used illicit drugs recently, in comparison to 4.3% who had self-reported recent drug use. There was little to no match between the results obtained by drug testing and self-reporting for each drug, and drug testing revealed that the most commonly used drugs were underreported. The analytical detection of a substance is considered reliable, as it follows forensic standards.

Similar to our study, previous studies obtained a higher drug use prevalence through biological testing (oral fluid sampling) compared to that through self-reporting. For example, studies conducted in Norway using oral fluid sampling revealed that 11% of music festival attendees tested positive for drug use, 6% self-reported recent drug use^9^, and among club goers in Oslo, 25% tested positive, while 14% self-reported recent drug use^6,26^. Furthermore, in a study conducted by our research group on a 36-h so-called party cruise departing from Sweden revealed that while 10% of participants tested positive for an illicit drug, only 4% self-reported drug use during the last 48 h^5^. In line with the present study, about 1% of participants declined drug testing, indicating that both oral fluid and breath sampling are well accepted among nightlife participants. The study also stated that in some cases, the sampling procedure using oral fluid was quite time consuming (> 15 min) due to mouth dryness, which was not a problem in the current study using the breath sampling device (procedure lasted 2–4 min). In the current study, participants were recruited at a club or festival (during the evening) and might have been in a greater hurry compared to participants on the 36-h party cruise-ship. This could be reflected by the 12 participants who dropped out before the breath test and highlights the need for fast sampling methods in such environments.

The present study identified the presence of 22 substances (related to 19 different substances) in the samples, most commonly the common central stimulants (cocaine, amphetamine, MDMA) and THC. Regarding the remaining drugs, only one to two participants tested positive. The same was observed in the Norwegian study among festival attendees, wherein 52 substances were tested, and with the exception of these four drugs and some benzodiazepines, few people tested positive for other substances^9^. Therefore, analyzing fewer substances such as common central stimulants might save costs, while still providing a good estimate of drug use prevalence in nightlife settings.

There was no to minimal agreement between drug test results and self-reports, which is in line with a study on cruise-ship^5^. In addition, no agreement was found for amphetamine and minimal agreement was found for MDMA and cocaine, which is in line with previous studies that found moderate agreement at best^3,9^. Although cannabis was the most commonly self-reported drug, the mismatch between the drug test and self-reported use was the largest for this substance. Nevertheless, THC was only detected among those who did not self-report recent use, indicating underreporting. Among participants who self-reported use during the last 48 h, none tested positive, which is most likely explained by the relatively short detection window of up to 6 h^36,37^. However, a comparative study revealed that concentrations of THC declined more rapidly in the oral fluid samples than in the breath samples of the same individuals during the 6 h of cannabis smoking that were measured^37^, indicating that the oral fluid sample is not a better alternative to the breath sample for the detection of cannabis use. Furthermore, a recent study has shown that the window of detection of THC in breath samples is similar to the window of impairment^38^.

Overall, the present study confirms previous studies demonstrating that drug use is often underreported. Possible reasons for underreporting could be a recall bias, a substance other than the one intended has been consumed or that drug consumption is regarded as socially unacceptable^39^.

Significantly more men than women tested positive for illicit drugs. Among those who tested positive, 21% of men and 6% of women self-reported recent drug use. Together, these results indicate that women use drugs less often and underreport them to a greater extent. In fact, a study among youth health clinics in Sweden demonstrated that girls in treatment for substance use disorder have more serious symptoms and problems than boys, and the authors hypothesized that girls receive help at a later stage and do not receive adequate treatment in time^40^. These findings might reflect a reduced societal acceptance of drug use among females but also highlight the importance of including women in preventive interventions.

Similar to previous studies that linked hazardous alcohol consumption with drug use^4,5,9,10,41^, the present study showed that BAC levels were higher among those who tested positive for illicit drugs. Therefore, there is a need for preventive interventions that target drug use and hazardous drinking in nightlife settings. Examples of such interventions are the multicomponent programs 'Clubs against Drugs' and 'Responsible Beverage Service.' These programs were associated with a number of positive outcomes such as increased refusal rates of drug-intoxicated guests to enter licensed premises or alcohol-intoxicated or underaged guests to serve alcohol, reduced observed drug use prevalence among guests, and reduced police-reported violence^42–48^. Based on the present results, future programs should focus on addressing both alcohol and drug use together and detect drug use early among women.

### Strengths and limitations

The strengths of the current study are the relatively large number of participants and the collected biological data in the form of breath samples to measure illicit drug use. Another strength is the method of chemical analysis used, which has been extensively tested, follows forensic standards and is thereby considered reliable. A limitation of the study might be a potential selection bias due to the fact that participation is voluntary. However, considering the level of underreporting, such a bias might likely be in the direction in which drug use is higher among those declining participation. Similarly, among participants, the proportion of men was smaller than that among non-participants, potentially presenting a selection bias. Considering that men more often tested positive for drug use than women, this bias is also likely in the direction of higher drug use among non-participants. As age was only estimated for non-participants, it is not clear if the two-year difference displays another selection bias or measurement error nor is it clear what an age bias would mean, as we have recently shown that the prevalence of last-year drug use among Swedish nightlife participants is not linear with age and is dependent on the substance used^49^.

The rate of participation was lower than that in the cruise-ship study^5^ but similar to the Norwegian nightlife studies mentioned above^9,26^. In line with studies using oral fluid sampling, refusal to breath sampling was generally low (1%)^5,9,26^. Furthermore, drug use prevalence might differ between locations and countries and, consequently, the willingness to be drug tested or declare drug use; therefore, future studies at different locations are needed. Drug prevalences might be difficult to compare across different specimens sampled as the detection time could vary. For example, whereas the detection time for tramadol was longer in breath than blood samples^50^, the opposite was true for THC^51^. Therefore, more studies comparing the detectability of drugs using different specimens are needed.

## Conclusions

The present study demonstrates that sampling exhaled breath is a feasible method to investigate illicit drug use among a large number of nightlife participants in field conditions. This method can be used to estimate the prevalence of illicit drug use, including cocaine, amphetamine, and MDMA (ecstasy) during the last 48 h as well as the use of cannabis with a shorter detection time. Furthermore, estimating drug use prevalence using this method is more reliable than self-reporting. Few participants refused breath sampling, and the briefness of the procedure leaves enough time for alcohol measurement and a variety of interview questions. Breath tests can be a valuable tool to investigate substance use among a large number of people in various settings, such as different nightlife settings or roadside testing^33^, as well as a pre- and post-prevalence measurement to assess the effects of preventive interventions.

## Methods

A cross-sectional study using breath sampling to measure drug use was conducted at five nightclubs in Stockholm and at a large two-day music festival (approximately 65,000 attendees) in Stockholm during the summer and fall of 2018. Data were collected in the evening until the closing time of the venue. Data collection was conducted only upon the event organizers' approval. The study was approved by the Regional Ethical Review Board of Stockholm (2017/1207–32) and all methods were performed in accordance with the relevant guidelines and regulations.

### Procedures

**Figure 1:**
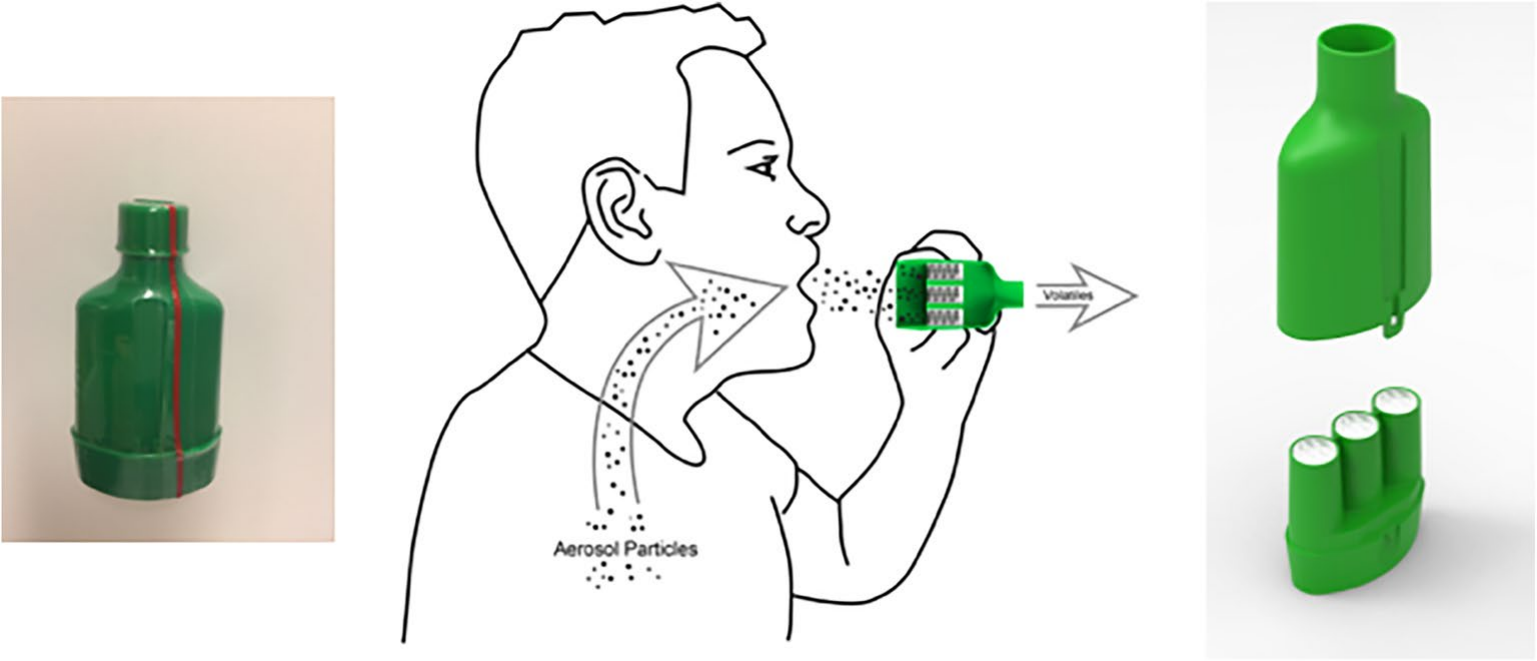
The BreathExplor device used for breath sampling (Munkplast, Uppsala). Before use, the plastic foil and inlet and outlet cups were removed (left). Participants were instructed to exhale 10 times through the device inlet. The device contained filters that collected aerosol particles from exhaled breath (middle). In the laboratory, microparticles were extracted from the filters and analyzed for the presence of illicit drugs using liquid chromatography and mass spectroscopy (right). The drawing was kindly provided by Munkplast.

The procedure was similar to that used in various nightlife settings^5,6,25,52^. At each event, the researcher was responsible, and data collection was conducted by one or two teams. Each team consisted of four to seven research assistants acting as recruiters or interviewers. Research assistants were trained by the researchers for both roles. Training included how to approach participants, informed consent, mouth rinsing before breath measurements, the interview process, as well as how to correctly handle the drug breath sampling device (Breath Explor, Munkplast^34^, Uppsala, Sweden, Fig. 1) and the breathalyzer (Dräger Alcotest 6820, Sollentuna, Sweden) to estimate blood alcohol concentration (BAC) level. Recruiters approached every third person passing an imaginary line located where there was a flow of people and invited them to participate in an alcohol and drug study^4,5^. Participation in the study was anonymous and voluntary. If the approached person was part of a group, the whole group was invited to participate in order to reduce refusal rates^53^. Upon refusal, the age and sex of the approached individuals were subjectively estimated.

Individuals who agreed to participate were directed to the interviewer. Informed consent was obtained from all participants. The participants were given a cup of water to rinse their mouths. The interview contained questions on demographics (age, gender, and occupation) and the participants’ smoking (tobacco) and alcohol drinking habits (alcohol use disorder identification test-C, AUDIT-C)^54^. The participant's BAC level was measured using a breathalyzer. If asked for, the interviewer gave feedback on the participant’s BAC level, and contact information to an alcohol helpline was provided if needed. The participants were then asked to complete a questionnaire regarding their use of different types of illicit drugs during their lifetime and the past 48 h. At the end of the interview, the participant was asked to exhale 10 times through the drug breath sampling device’s inlet (Fig. 1), in accordance with the manual. To enable matching between the questionnaire and the drug breath sampling device, each was marked by the researchers using a sticker containing an identical code. The breath sampling devices were stored in cooler boxes (4 °C) during data collection, and then stored at − 20 °C until further chemical analysis. The participants were informed that no drug test results could be observed onsite. Participants could choose a small incentive (e.g., chips, chocolate bar, chewing gum).

### Chemical analysis

Frozen breath sampling devices were transported to the laboratory in cooler boxes. One of the three collectors (Fig. 1) was removed from the sampling device for analysis, and the microparticles were extracted using a methanol solution containing internal standards. Samples were analyzed as previously published^34,35^ using a combination of liquid chromatography and tandem mass spectroscopy with electrospray ionization and selective reaction monitoring. This method has high sensitivity and specificity, with a detection limit of 1 pg/collector. In total, 47 different substances were analyzed in each sample (Table 4). The detection time limit for the substances analyzed was estimated to be between 24 and 48 h after consumption, with the exception of THC, for which the detection window is shorter^33,36,37,55^. Positive test results for metabolites have been treated as an indication for the illicit drug (see Table 4): ritalinic acid for methylphenidate; EDDP for methadone; norbuprenorphine for buprenorphine; benzoylecgonine for cocaine; and morphine, codeine, and 6-acetylmorphine for heroin^56^.

**Table 4:** List of substances analyzed in the breath samples.

Cannabis (THC)	**Central stimulants**
Ketamine	Cocaine
**Opioids**	Benzoylecgonine (Cocaine metabolite)
*Heroin metabolites*	Amphetamine
6-Acetylmorphine	Methamphetamine
Morphine	Ecstasy (MDMA)
6-Acetylcodeine	Mephedrone (4-MMC)
Codeine	MDA
*Pain medications*	MDPV
Dihydrocodeine	4-Methylcathinone
Hydromorphone	Methylone (MDMC)
Tramadol	Butylone
O-Desmethyltramadol	PMMA
Oxycodone	*Medications for ADHD*
*Medications for opioid use disorder*	Methylphenidate/Ritalin
Methadone	Ritalinic acid (Methylphenidate metabolite)
EDDP (methadone metabolite)	**New Psychoactive Substances (NPS)**
Buprenorphine	Alpha-PVP
Norbuprenorphine (Buprenorphine metabolite)	Pentedrone
**Benzodiazepines**	MDEA
Diazepam	MBDB
Nordazepam	BDB (MBDB metabolite)
Oxazepam	**Other medications (classed as narcotics)**
Temazepam	Zopiclone
Flunitrazepam	Zolpidem
Alprazolam	Pregabalin
Bromazepam	Gabapentin
Midazolam	
Lorazepam	

Alpha-PVP: alpha-pyrrolidinovalerophenone, BDB: 1,3-Benzodioxolylbutanamine, EDDP: 2-Ethylidene-1,5-dimethyl-3,3-diphenylpyrrolidine, MBDB: 1,3-Benzodioxolyl-N-methylbutanamine, MDA: 3,4-Methylenedioxyamphetamine, MDEA: 3,4-Methylenedioxy-N-ethylamphetamine, MDMA: 3,4-Methylenedioxymethamphetamine, MDMC:3,4-methylenedioxy-*N*-methylcathinone, MDPV: Methylenedioxypyrovalerone, 4-MMC: 4-methyl methcathinone, PMMA: para-Methoxyamphetamine, THC: Tetrahydrocannabinol.

### Statistical analysis

Data were analyzed using SPSS (version 27). Chi-squared (χ^2^)-analysis was conducted to compare proportions of tested drug use across genders as well as to compare the distribution of gender among participants and those denying participation. The Mann–Whitney U test was used to compare BAC levels between the participants who tested positive and negative for illicit drug use. The Mann–Whitney U-test was used to compare the distribution of age (skewed distribution) between participants and people denying participation. The level of agreement between self-reported data and biological test results were measured with Cohen’s kappa (κ) and were defined as follows: no agreement, ≤  0.20; minimal, 0.21–0.39; weak, 0.40–0.59; moderate, 0.60–0.79; strong, 0.80–0.90; and almost perfect, > 0.90^57^. BAC levels were presented in volume percent (i.e., 1 g ethanol per 100 mL blood). The significance level alpha was set at 0.05. When including gender in the tests, participants who reported “other” (n = 4) were excluded.

### Ethical approval

The study was approved by the Regional Ethical Review Board of Stockholm (2017/1207–32). Prof. Olof Beck was an advisor to Munkplast in the role of scientific expert. Sigurd Hermansson is an employee of Waters Corporation.

## Data Availability

Data will be available upon request. Requests can be sent to Kristin.Feltmann@ki.se.
